# Fusarochromanone-induced reactive oxygen species results in activation of JNK cascade and cell death by inhibiting protein phosphatases 2A and 5

**DOI:** 10.18632/oncotarget.5996

**Published:** 2015-10-17

**Authors:** Ying Gu, Mansoureh Barzegar, Xin Chen, Yang Wu, Chaowei Shang, Elahe Mahdavian, Brian A. Salvatore, Shanxiang Jiang, Shile Huang

**Affiliations:** ^1^ Laboratory of Veterinary Pharmacology and Toxicology, College of Veterinary Medicine, Nanjing Agricultural University, Nanjing, Jiangsu Province, P. R. China; ^2^ Department of Biochemistry and Molecular Biology, Louisiana State University Health Sciences Center, Shreveport, LA, USA; ^3^ Feist-Weiller Cancer Center, Louisiana State University Health Sciences Center, Shreveport, LA, USA; ^4^ Department of Chemistry and Physics, Louisiana State University, Shreveport, LA, USA

**Keywords:** fusarochromanone, cell death, reactive oxygen species, JNK, protein phosphatase 2A

## Abstract

Recent studies have shown that fusarochromanone (FC101), a mycotoxin, is cytotoxic in a variety of cell lines. However, the molecular mechanism underlying its cytotoxicity remains elusive. Here we found that FC101 induced cell death in COS7 and HEK293 cells in part by activating JNK pathway. This is evidenced by the findings that inhibition of JNK with SP600125 or expression of dominant negative c-Jun partially prevented FC101-induced cell death. Furthermore, we observed that FC101-activated JNK pathway was attributed to induction of reactive oxygen species (ROS). Pretreatment with N-acetyl-L-cysteine (NAC), a ROS scavenger and antioxidant, suppressed FC101-induced activation of JNK and cell death. Moreover, we noticed that FC101 inhibited the serine/threonine protein phosphatases 2A (PP2A) and 5 (PP5) in the cells, which was abrogated by NAC. Overexpression of PP2A or PP5 partially prevented FC101-induced activation of JNK and cell death. The results indicate that FC101-induced ROS inhibits PP2A and PP5, leading to activation of JNK pathway and consequently resulting in cell death.

## INTRODUCTION

Fusarochromanone (FC101) is a mycotoxin produced by food-borne fungi such as *Fusarium equiseti* (*F. equiseti*) and *F. roseum*. The wide presence and persistence of this mycotoxin in the food chain may lead to an inevitable exposure to humans and animals [1–4]. The contamination of FC101 in the feedstuffs was first discovered to cause avian tibial dyschondroplasia (ATD) in broiler chickens, a disease characterized by bone deformation [2, 5–8]. Later, FC101 was also suspected to be associated with etiology of Kashin-Beck disease (a chronic and endemic osteochondropathy) in children in northeastern and southwestern China, as well as in southeastern Siberia and northern Korea [9, 10]. Furthermore, FC101 has recently been found to inhibit cell proliferation and differentiation as well as induce cell death in lymphocytes, osteoblasts, melanoma cells, breast cancer cells, glioblastoma cells, normal cardiac fibroblasts and kidney cells [11–16]. Despite these findings, how FC101 causes cytotoxicity is not well understood.

Studies have demonstrated that the toxicity of many mycotoxins is often related to induction of oxidative stress, such as reactive oxygen species (ROS), in various types of cells [17–26]. Certain levels of ROS are critical for many physiological functions of all aerobic organisms [27]. However, excessive ROS may disturb metabolic pathways; deplete cellular antioxidants; damage DNA, proteins and lipids; and activate/inhibit related signaling pathways [27]. Consequently, this results in inhibition of cell differentiation or proliferation and even induction of cell death [27–30]. Recently, it has been noticed that FC101 can induce ROS in glioblastoma cells [14]. However, it is unclear whether and how FC101 exerts its cytotoxicity by induction of ROS.

Increasing evidence has implicated that the members of the mitogen activated protein kinase (MAPK) family play a critical role in the regulation of cell survival [31]. Particularly, in response to stress stimuli, e.g. oxidative stress, MAPKs can be activated, leading to apoptosis [29, 30]. There are at least three distinct sub-families of MAPKs, including the extracellular signal-regulated kinases Erk1/2, Erk3/4, Erk5, Erk7/8, the c-Jun N-terminal kinases JNK1/2/3, and the p38 MAPKs p38α/β/γ/δ in mammalian cells [31–33]. Activation of MAPKs requires phosphorylation of certain tyrosine, serine and/or threonine residues in the activation loops [31]. Phosphorylation of MAPKs is balanced by specific MAPK kinases and phosphatases [31, 32]. It has been demonstrated that MAPK phosphatase 1 (MKP-1) and protein phosphatase 2A (PP2A) negatively regulate Erk1/2, JNK and/or p38, whereas protein phosphatase 5 (PP5) negatively regulates JNK/p38 pathway [29, 30, 34–37]. Further, protein phosphatase 1 (PP1) can also negatively regulate both JNK and p38 MAPK [38]. PP2A, a serine and threonine protein phosphatase, is a heterotrimeric holoenzyme composed of a catalytic subunit (PP2Ac), an A regulatory subunit (PP2A-A, also termed PR65), and a number of B regulatory subunits (PP2A-B), including B (PR55), B' (PR61), B” (PR72) and B'” (PR93/PR110) [39]. The phosphatase activity of PP2Ac or PP5 is modulated by association with the regulatory subunits of PP2A-A and PP2A-B [34, 39]. Also, PP2A activity is mediated by the phosphorylation and methylation of PP2Ac [40, 41]. Accumulating data have shown that PP2A can negatively regulate the phosphorylation of Erk1/2, JNK and p38, whereas PP5 can negatively regulate the phosphorylation of JNK/p38 under stress conditions [29, 30, 34–37].

Here, for the first time, we show that FC101-induced ROS caused cell death, by activation of JNK cascade in COS7 and HEK293 cells. Furthermore, we identified that FC101-induced ROS activated JNK pathway, by inhibiting PP2A and PP5. Our findings suggest that the toxicity of FC101 in humans and animals may be prevented and treated by pharmacological interventions, such as antioxidants and JNK inhibitors.

## RESULTS

### FC101 reduces cell viability by induction of ROS

**Figure 1:**
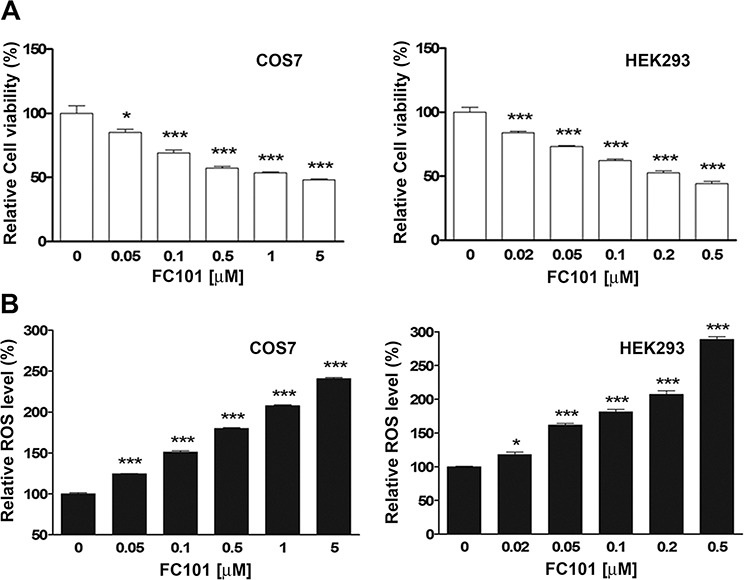
FC101 reduces cell viability and coincidently induces ROS in COS7 and HEK293 cells COS7 and HEK293 cells were exposed to FC101 at indicated concentrations for 24 h. (A) Cell viability was evaluated using one solution reagent. (B) ROS level was detected using CM-H2DCFDA. All data represent the means ± SE (*n* = 3). *P < 0.05, ***P < 0.001.

To determine whether FC101-induced cytotoxicity is related to induction of ROS, COS7 and HEK293 cells were treated with FC101 (0–5 μM) for 24 h, followed by assays for cell viability and ROS induction. As shown in Fig. 1A, treatment with FC101 for 24 h resulted in a concentration-dependent decrease of cell viability in both COS7 and HEK293 cells. The decreased cell viability was in agreement with the increased levels of ROS (Fig. 1B). The data imply that the induction of ROS by FC101 might be associated with the cytotoxicity in COS7 and HEK293 cells.

**Figure 2:**
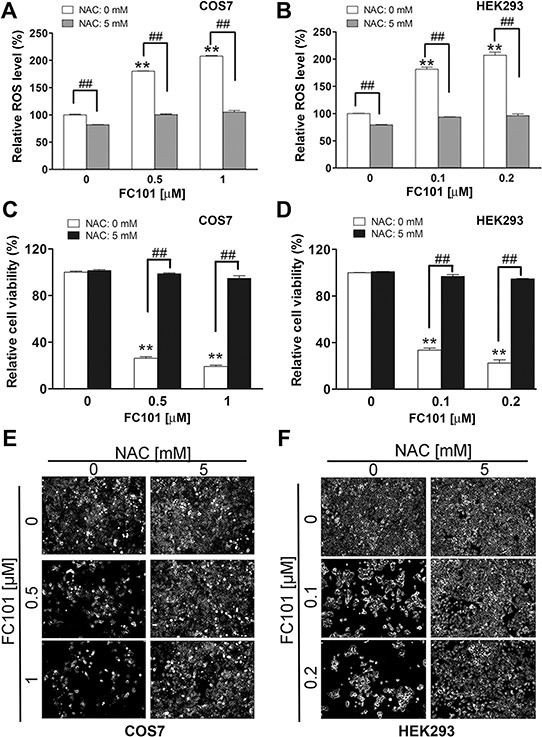
N-acetyl-L-cysteine (NAC) prevents FC101 from inducing ROS and cell death COS7 and HEK293 cells were pretreated with NAC (5 mM) for 1 h. (**A** and **B**) The Cells were then exposed to FC101 at indicated concentrations for 24 h, (**C** and **E**) for 6 days (for COS7), or (**D** and **F**) for 4 days (for HEK293), followed by ROS detection (A and B), cell viability assay (C and D), morphological analysis (E and F). All data represent the means ± SE (*n* = 3). *P < 0.05, **P < 0.01, ^##^*P* < 0.01.

To confirm whether FC101-induced cytotoxicity is indeed due to ROS induction, COS7 and HEK293 were pretreated for 1 h with 5 mM of NAC (a ROS scavenger and antioxidant), and then exposed to FC101 (0–1 μM) for 24 h (for ROS detection) or 4–6 days (for cell viability assay or morphological analysis). As expected, pretreatment with NAC strongly blocked FC101 induction of ROS in the cells (Fig. 2A and 2B). Also, NAC potently suppressed FC101-induced loss of cell viability in the cells (Fig. 2C-2F). Taken together, the findings indicate that FC101 induces cytotoxicity primarily through induction of ROS, which can be prevented by the ROS scavenger NAC.

### FC101 activates JNK by induction of ROS

**Figure 3:**
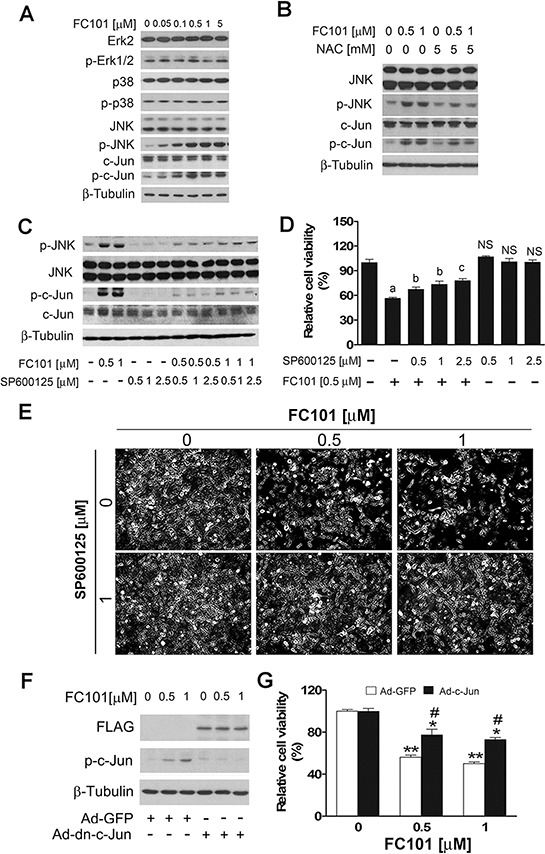
FC101-inducted ROS activates JNK cascade, leading to cell death (**A**) COS7 cells were exposed to FC101 at indicated concentrations for 24 h, (**B**) pretreated with NAC (5 mM) for 1 h, or (**C**) pretreated with SP600125 for 30 min, and then exposed to FC101 for 24 h, followed by Western blotting with indicated antibodies. Similar results were observed in at least 3 independent experiments. (**D** and **E**) COS7 cells were pre-treated with/without the JNK inhibitor SP600125 for 30 min, and then treated with/without indicated concentrations of FC101 for 24 h, followed by cell viability assay (D) and morphological analysis (E), respectively. (**F** and **G**) COS7 cells, infected with recombinant adenovirus encoding FLAG-tagged dominant negative c-Jun (Ad-dn-c-Jun) or GFP (control) for 24 h, were exposed to FC101 (0–1 μM) for 24 h, followed by Western blotting (F) and cell viability assay (G). All data represent the means ± SE (*n* = 3). NS, no significant difference, ^a^*P* < 0.01, difference with the control group, ^b^*P* < 0.05, ^c^*P* < 0.01, difference with the 0.5 μM FC101 group, *P < 0.05, **P < 0.01, difference with the control group. ^#^*P* < 0.05, the Ad-dn-c-Jun group vs the Ad-GFP group.

Since MAPKs play a crucial role in ROS-induced cell death [29–31], we next investigated whether FC101-induced cell death is associated with activation of Erk1/2, p38 and JNK. As shown in Fig. 3A, treatment with FC101 for 24 h did not obviously alter the phosphorylation of Erk1/2 and p38, but induced the phosphorylation of JNK in a concentration-dependent manner in COS7 cells. Noticeably, FC101 activation of JNK also resulted in a robust phosphorylation of c-Jun, a substrate of JNK (Fig. 3A). Furthermore, pretreatment with NAC (5 mM) for 1 h blocked FC101-induced phosphorylation of JNK and c-Jun in the cells (Fig. 3B). The results suggest that FC101 activates JNK pathway by induction of ROS.

To determine the role of JNK in FC101-induced cytotoxicity, COS7 cells were pretreated with or without SP600125 (JNK inhibitor) for 30 min, and then exposed to FC101 for 24 h. As shown in Fig. 3C, pretreatment with SP600125 (0.5–2.5 μM) profoundly blocked FC101-induced phosphorylation of c-Jun, as the surrogate of JNK activity. As the JNK inhibitor was able to inhibit JNK/c-Jun phosphorylation induced by FC101, we next examined whether the JNK inhibitor attenuates FC101-induced cytotoxicity. For this, COS7 cells were pretreated with or without SP600125 for 30 min, followed by exposure to FC101 (0.5 or 1 μM) for 24 h. As shown in Fig. 3D and 3E, 0.5–2.5 μM of SP600125 itself did not obviously influence the basal cell viability, but significantly prevented FC101 from reducing cell viability. Similar results were also seen in the HEK 293 cells (data not shown). In addition, we further confirmed the above finding by genetic manipulation. Infection of COS7 with Ad-dn-c-Jun resulted in expression of FLAG-tagged dominant negative c-Jun (dn-c-Jun), as detected by Western blotting with antibodies to FLAG (Fig. 3F). Expression of the dn-c-Jun attenuated FC101-reduced cell viability in COS7 cells (Fig. 3G). Collectively, our data indicate that FC101-induced cytotoxicity in COS7 and HEK 293 cells is at least in part via activating JNK pathway.

### FC101 induction of ROS inhibits PP2A and PP5

**Figure 4:**
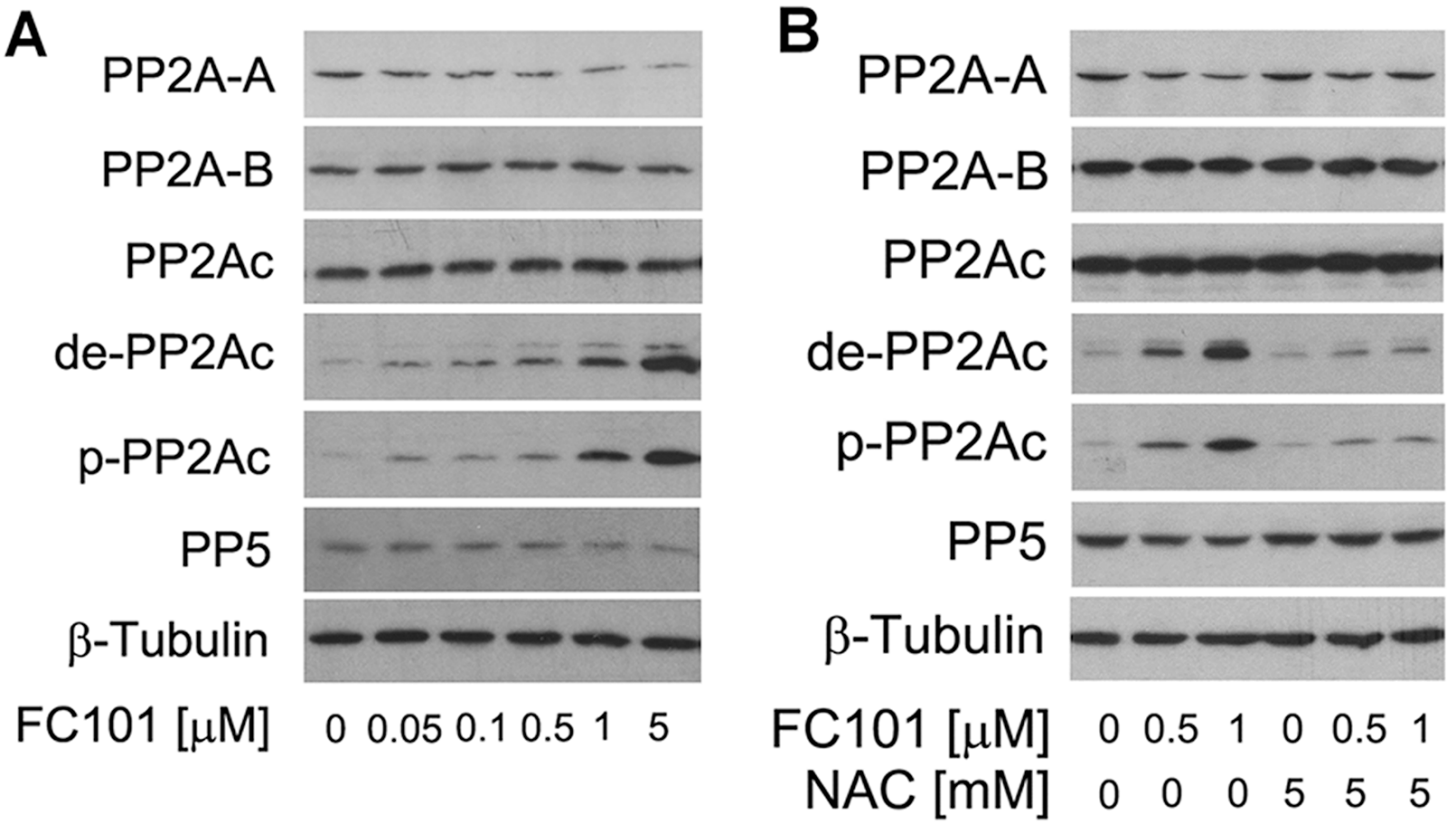
FC101 induction of ROS downregulates PP2A and PP5 (**A**) COS7 cells were treated with 0–5 μM FC101 for 24 h, or (**B**) pretreated with/without NAC (5 mM) for 1 h, and then exposed to FC101 (0–1 μM) for 24 h, followed by Western blot analysis with indicated antibodies. Similar results were observed in at least 3 independent experiments.

It has been demonstrated that MKP-1 and PP2A negatively regulate Erk1/2, JNK and/or p38, whereas PP1 and PP5 negatively regulate JNK/p38 [29, 30, 34–38]. In the present study, since FC101 was found to induce activation of JNK, but not Erk1/2 and p38 MAPK (Fig. 3A), we deduced that MKP-1 and PP1 were not inhibited by FC101. Therefore, we reasoned that FC101 might activate the JNK pathway by inhibiting PP2A and/or PP5. To this end, COS7 cells were exposed to 0–5 μM of FC101 for 24 h, followed by Western blotting. As shown in Fig. 4A, FC101 did not apparently alter the cellular protein level of the catalytic subunit PP2Ac, but increased the expression of demethylated- and phospho-PP2Ac, two events related to inhibition of PP2A activity [39], concentration-dependently. Also, FC101 did not affect the expression of PP2A-B, but reduced the protein levels of PP2A-A and PP5 in a concentration dependent manner (Fig. 4A). The results suggest that FC101 inhibits PP2A and PP5.

Since FC101-induced ROS activated the JNK pathway (Fig. 3), we next wondered whether FC101 inhibition of PP2A/PP5 is associated with induction of ROS. For this, COS7 cells were pretreated with or without NAC for 1 h, and then exposed to FC101 for 24 h. By Western blot analysis, we found that NAC potently blocked FC101-induced demethylated- and phospho-PP2A, and prevented FC101-induced down-regulation of PP2A-A and PP5 in the cells (Fig. 4B). Similar data were seen in the HEK293 cells (data not shown). Our findings imply that FC101-induced ROS might inhibit PP2A and PP5, resulting in activation of JNK.

### Overexpression of PP2A and PP5 partially prevents FC101-induced JNK activation and cell death

**Figure 5:**
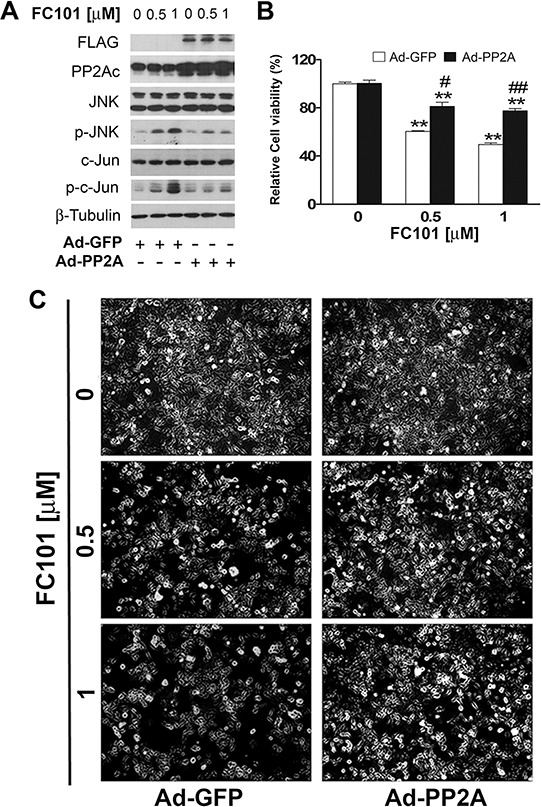
Overexpression of PP2A partially prevents FC101-induced JNK activation and cell death (**A-C**) COS7 cells infected with Ad-PP2A and Ad-GFP (as control) were exposed to the indicated concentrations of FC101 for 24 h, followed by Western blot analysis with indicated antibodies (A), morphological analysis (B), and cell viability assay (C). Similar results shown in (A and B) were observed in at least 3 independent experiments. All data shown in (C) represent the means ± SE (*n* = 3). *P < 0.05, **P < 0.01, difference with the control group, ^#^*P* < 0.05, ^##^*P* < 0.01, the Ad-PP2A group vs the Ad-GFP group.

**Figure 6:**
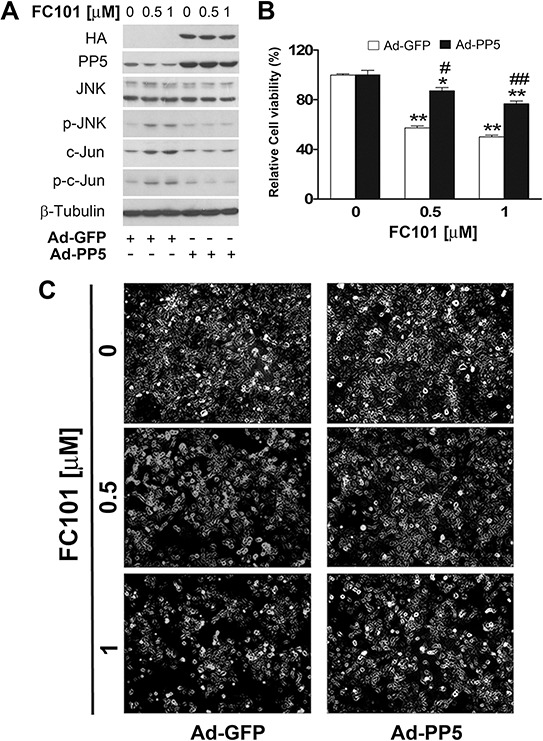
Overexpression of PP5 partially prevents FC101-induced activation of JNK as well as cell death (**A-C**) COS7 cells, infected with Ad-PP5 or Ad-GFP (as control), were exposed to exposed to indicated concentrations of FC101 for 24 h, followed by Western blot analysis with indicated antibodies (A), morphological analysis (B), and cell viability assay (C). Similar results shown in (A and B) were observed in at least 3 independent experiments. All data shown in (C) represent the means ± SE (*n* = 3). *P < 0.05, **P < 0.01, difference with the control group, ^#^*P* < 0.05, ^##^*P* < 0.01, the Ad-PP5 group vs the Ad-GFP group.

To verify the significance of PP2A and PP5 in FC101-induced activation of JNK and cell death, we further studied whether overexpression of PP2A or PP5 impacts FC101 in activation of JNK and cytotoxicity. For this, COS7 cells, infected with Ad-PP2A, Ad-PP5 or Ad-GFP (as control), were exposed to FC101 (0.5 and 1 μM) for 24 h, followed by Western blotting, morphological analysis and cell viability assay. We observed that overexpression of PP2A remarkably attenuated FC101-induced activation of JNK (Fig. 5A) as well as cell cytotoxicity (Fig. 5B and 5C). Similarly, overexpression of PP5 also potently prevented FC101-induced cell cytotoxicity by inactivating JNK (Fig. 6A-6C). Together, our results support the notion that FC101 induces cytotoxicity at least in part by inhibiting PP2A and PP5, leading to activation of JNK pathway.

## DISCUSSION

Reactive oxygen species (ROS) generated from both endogenous and exogenous insults play a critical role in the toxic injury process of cells [27, 42]. Recent studies have demonstrated that the toxicity of a variety of mycotoxins, such as zearalenone, T-2 toxin, ochratoxin A, fumonisin B1, aflatoxin B, and patulin, is related to induction of ROS in various types of cells [17–26]. FC101 is a toxic fungal metabolite mainly produced by *F. equiseti* [12], which has been observed frequently in human food and animal feedstuffs [1–4, 43]. Although it has been shown that FC101 induces cell death [11–16], the underlying molecular mechanism is still poorly understood. Here we demonstrate that FC101 induces ROS, causing the activation of the stress kinase JNK cascade, leading to cell death. Our findings suggest that the toxicity of FC101 in humans and animals may be prevented and treated by pharmacological interventions, such as antioxidants and JNK inhibitors.

Recently, it has been observed that FC101 increases the level of hydrogen peroxide, a kind of ROS, in glioblastoma cells (A172) [14]. However, whether the induction of hydrogen peroxide contributes to the cell death is not known. In the present study, we found that FC101 increased the levels of ROS in both COS7 and HEK293 cells, although the two cell lines exhibited a slightly different sensitivity to FC101 (Fig. 1). Interestingly, the cell viability reduction by FC101 was closely related to the level of ROS induced. More importantly, NAC, a ROS scavenger, was able to almost completely block FC101-induced cell death (Fig. 2). Therefore, our data strongly support the notion that FC101, like many other mycotoxins mentioned above, induces cell death predominantly by induction of ROS.

Here we found that FC101-induced ROS activated JNK cascade, leading to cell death in HEK293 and COS7 cells, which is, to some extent, different from the findings in other mycotoxins [44–46]. For instance, it has been shown that satratoxin H, a mycotoxin, induces apoptosis of PC12 cells through the activation of JNK and p38 MAPK due to ROS induction [44]. Similarly, aflatoxin G1-induced ROS triggers apoptosis in A549 cells by activating JNK and p38 pathways [45]. Exposure to T-2 toxin induces ROS and the phosphorylation of Erk1/2, p38 MAPK and JNK, causing apoptosis in human neuroblastoma cells (IMR-32) [46]. Therefore, the above findings indicate that many mycotoxins can induce cell death by induction of ROS, but the signaling pathways targeted apparently differ from case to case.

In the present study, we provide evidence that FC101-induced cell death is due to ROS-mediated inhibition of PP2A and PP5, leading to activation of JNK cascade. This is supported by the following observations. Firstly, FC101 induced ROS, leading to cytotoxicity of COS7 and HEK293 cells. This process could be almost completely prevented by NAC (Fig. 2), a ROS scavenger, indicating that the induction of ROS contributes to FC101-induced cytotoxicity. Secondly, FC101 induced activation of JNK, but not Erk1/2 and p38, resulting in the cytotoxicity, which was markedly attenuated by SP600125 (JNK inhibitor) or by expression of dominant negative c-Jun (Fig. 3). This suggests that among the MAPKs, only JNK is involved in FC101-induced cytotoxicity. Thirdly, activation of the JNK signaling by FC101 was remarkably inhibited by NAC (Fig. 3), revealing that FC101 activation of JNK is related to its induction of ROS. Fourthly, FC101-induced ROS obviously increased the expression of demethylated- and phospho-PP2Ac (Fig. 4), two events related to inhibition of PP2A [39], and reduced the protein level of PP5 (Fig. 4), directly related to inhibition of PP5. Furthermore, FC101-induced ROS also decreased the protein level of PP2A-A (Fig. 4), a regulatory subunit shared by both PP2A and PP5 [34, 39], indicating inhibition of both PP2A and PP5. Lastly, overexpression of PP2A or PP5 prevented FC101-induced activation of JNK as well as cell death (Fig. 5 and 6). To our knowledge, this is the first study to unveil how FC101 induces cell death by mediating PP2A/PP5-JNK signaling pathway.

However, at this stage, we do not know how FC101 induction of ROS increases the expression of demethylated-PP2Ac and phospho-PP2Ac (Tyr307), leading to inhibition of PP2A activity. PP2Ac is uniquely methylated on Leu309 by the leucine carboxyl methyltransferase-1 (LCMT-1) [47, 48], and demethylated by protein phosphatase methylesterase-1 (PME-1) [49]. Besides, the phosphorylation of PP2Ac on Tyr307 can be triggered by activation of SRC kinase or epidermal growth factor receptor (EGFR), and be eliminated by protein tyrosine phosphatase 1B (PTP1B) [50]. Further studying the effects of FC101 on the activity of LCMT-1, PME-1, SRC, EGFR and/or PTP1B may clarify how PP2A is inhibited by FC101-induced ROS. In addition, currently we have no clue as to how PP5 protein level was downregulated by FC101-induced ROS. It has been reported that cadmium-induced ROS or hydrogen peroxide can also downregulate PP5 protein expression in neuronal cells [29, 30]. These findings suggest that oxidative stress-induced downregulation of PP5 protein level may be a general feature. Identifying how PP5 is downregulated by ROS may be helpful for design of new interventions against oxidative stress-induced cytotoxicity or diseases.

A new question arising from the current study is how FC101 increases the level of ROS in the cells. Given that the level of ROS in a cell is tightly regulated by the ROS generation system and the ROS clearance system [51], it would be interesting to determine whether the elevated level of ROS is attributed to increased generation of ROS by activating NADPH oxidases and/or decreased clearance of ROS by inhibiting superoxide dismutase or catalase in the cells. The new data may be instructive for the development of more effective strategy against the toxicity of FC101 clinically in humans and animals.

In summary, here we show that FC101-inducd cell death was attributed to the induction of ROS in COS7 and HEK293 cells. Mechanistically, FC101-induced ROS was able to suppress the activity of PP2A and PP5, which resulted in activation of the stress kinase JNK, thereby leading to cell death. Our findings shed a new insight on the molecular mechanism of FC101's cytotoxicity.

## MATERIALS AND METHODS

### Materials

Fusarochromanone (FC101) was isolated and purified (purity >97%, by NMR) from rice cultures of the fungus *F. equiseti*, and was converted into the stable and water-soluble phosphate-salt form, as described [12]. The phosphate-salt form of FC101 was then dissolved in Milli-Q water and filtered through a 0.2 μm syringe filter to prepare a sterile stock solution (5 mM), aliquoted and stored at −20°C. Dulbecco's modified Eagle's medium (DMEM), MEM non-essential amino acids and 0.05% trypsin-EDTA were obtained from Mediatech (Herndon, VA), fetal bovine serum (FBS) from Atlanta Biologicals (Lawrenceville, GA), enhanced chemiluminescence solution from Perkin-Elmer Life Science (Boston, MA), CellTiter 96^®^ AQ_ueous_ One Solution Cell Proliferation Assay kit from Promega (Madison, WI), SP600125 from LC Laboratories (Woburn, MA), 5-(and-6)-chloromethyl-2′,7′-dichlorodihydrofluorescein diacetate (CM-H_2_DCFDA) from Invitrogen (Carlsbad, CA).

### Cell lines and cultures

Both African green monkey kidney fibroblast-like cells (COS7) and human embryonic kidney 293 cells (HEK293) were purchased from American Type Culture Collection (Manassas, VA), and were grown in antibiotic-free DMEM supplemented with 10% FBS and 1% MEM non-essential amino acids. All cells were cultured in a humid incubator (37°C and 5% CO_2_).

### Recombinant adenoviral constructs and infection

The recombinant adenoviruses encoding hemagglutinin (HA)-tagged wild-type (wt) human PP5 (Ad-PP5), FLAG-tagged wt rat PP2ACα (Ad-PP2A), FLAG-tagged dominant negative (dn) c-Jun (FLAG-Δ169) (Ad-dn-c-Jun), and GFP (Ad-GFP) were described previously [52, 53]. For experiments, cells were grown in the growth medium and infected with individual adenovirus for 24 h at 5 of multiplicity of infection (MOI = 5). Subsequently, cells were used for experiments. Ad-GFP served as a control. Overexpression of FLAG-tagged dn-c-Jun or wt-PP2A and HA-tagged wt-PP5 was confirmed by Western blotting with antibodies to FLAG and HA, respectively.

### Cell viability assay

Cell viability was evaluated using CellTiter 96^®^ AQ_ueous_ One Solution Cell Proliferation Assay kit (Promega), as described [16]. Briefly, cells suspended in the growth medium were seeded in a 96-well plate at a density of 1 × 10^4^ cells/well (in triplicates) and were grown overnight at 37°C in a humidified incubator with 5% CO_2_. The next day, cells were treated with FC101 (0–5 μM) for 24 h or with/without FC101 (0.5 μM) following pre-incubation with JNK inhibitor SP600125 (0.5, 1 and 2.5 μM) for 30 min, respectively. Additionally, cells, infected with Ad-GFP, Ad-c-Jun, Ad-PP2A and Ad-PP5, respectively, were exposed to FC101 (0–1 μM). After incubation for 24 h, each well was added with 20 μL of one solution reagent and was incubated for 1 h. Cell viability was determined by measuring the optical density (OD) at 490 nm using a Wallac 1420 Multilabel Counter (PerkinElmer Life Sciences, Wellesley, MA).

### Cell morphological analysis

Cells were seeded at a density of 2 × 10^5^ cells/well in a 6-well plate. The next day, cells were treated with FC101 (0–1 μM), following pre-incubation with/without SP600125 (1 μM) for 30 min. In some cases, after infection with Ad-PP2A, Ad-PP5 and Ad-GFP, respectively, cells were exposed to FC101 (0–1 μM) for 24 h. Additionally, cells were seeded at a density of 2 × 10^4^ cells/well in a 6-well plate. The next day, the cells were exposed to FC101 (0–1 μM) for 4–6 days following pretreatment with or without NAC (5 mM) for 1 h. Finally, images were taken with an Olympus inverted phase-contrast microscope equipped with the Quick Imaging system.

### ROS detection

The ROS level was measured by using CM-H_2_DCFDA, as described [54]. Briefly, cells were seeded at a density of 1 × 10^4^ cells/well in 96-well plates. The next day, cells were loaded with 10 μM CM-H_2_DCFDA following the manufacturer's protocol, and were incubated in the presence of FC101 (0–5 μM) for 24 h with triplicates of each treatment. In some cases, after loading with 10 μM CM-H_2_DCFDA for 40 min, cells were pre-incubated with NAC (5 mM) for 1 h and then treated with FC101 (0–1 μM) for 24 h. Fluorescent intensity was recorded by excitation at 485 nm and emission at 535 nm using a Wallac 1420 Multilabel Counter (PerkinElmer Life Sciences).

### Western blot analysis

Western blotting was performed as described previously [16]. The following antibodies were used: phospho-Erk1/2 (Thr202/Tyr204), phospho-p38 (Thr180/Tyr182), phospho-JNK (Thr183/Tyr185) (Cell Signaling Technology, Beverly, MA), PP2ACα and PP5 (BD Biosciences), PP2A-A subunit and PP2A-B subunit (Millipore, Billerica, MA), phospho-PP2A (Tyr307) (Epitomics, Burlingame, CA), JNK1 and c-Jun, phospho-c-Jun (Ser63), Erk2, p38, demethylated-PP2A and HA (Santa Cruz Biotechnology), FLAG and β-tubulin (Sigma), goat anti-mouse IgG-horseradish peroxidase and goat anti-rabbit IgG-horseradish peroxidase (Pierce, Rockland, IL).

### Statistical analysis

Results were expressed as mean values ± standard error (mean ± S.E.). Statistical analysis was performed using student *t*-test or one-way analysis of variance (ANOVA) followed by post hoc Dunnett's test for multiple comparisons. A level of *P* < 0.05 was considered to be significant.

## References

[1] Abbas HK, Mirocha CJ, Gunther R (1989). Mycotoxins produced by toxic Fusarium isolates obtained from agricultural and nonagricultural areas (Arctic) of Norway. Mycopathologia.

[2] Krogh P, Christensen DH, Hald B, Harlou B, Larsen C, Pedersen EJ, Thrane U (1989). Natural occurrence of the mycotoxin fusarochromanone, a metabolite of Fusarium equiseti, in cereal feed associated with tibial dyschondroplasia. Appl Environ Microbiol.

[3] Mirocha CJ, Abbas HK, Kommedahl T, Jarvis BB (1989). Mycotoxin production by Fusarium oxysporum and Fusarium sporotrichioides isolated from Baccharis spp. from Brazil. Appl Environ Microbiol.

[4] Xie WP, Mirocha CJ, Pawlosky RJ, Wen YC, Xu XG (1989). Biosynthesis of fusarochromanone and its monoacetyl derivative by Fusarium equiseti. Appl Environ Microbiol.

[5] Lee YW, Mirocha CJ, Shroeder DJ, Walser MM (1985). TDP-1, a toxic component causing tibial dyschondroplasia in broiler chickens, and trichothecenes from Fusarium roseum ‘Graminearum’. Appl Environ Microbiol.

[6] Walser MM, Morris VC, Levander OA (1988). Effect of dietary selenium on the development of Fusarium-induced tibial dyschondroplasia in broiler chickens. Avian Dis.

[7] Wu W, Cook ME, Chu Q, Smalley EB (1993). Tibial dyschondroplasia of chickens induced by Fusarochromanone, a mycotoxin. Avian Dis.

[8] Orth MW, Cook ME (1994). Avian tibial dyschondroplasia: a morphological and biochemical review of the growth plate lesion and its causes. Vet Pathol.

[9] Peng A, Yang C, Rui H, Li H (1992). Study on the pathogenic factors of Kashin-Beck disease. J Toxicol Environ Health.

[10] Sudre P, Mathieu F (2001). Kashin-Beck disease: from etiology to prevention or from prevention to etiology?. Int Orthop.

[11] Furmanski BD, Dréau D, Wuthier RE, Fuseler JW (2009). Differential uptake and selective permeability of fusarochromanone (FC101), a novel membrane permeable anticancer naturally fluorescent compound in tumor and normal cells. Microsc Microanal.

[12] Dréau D, Foster M, Hogg M, Culberson C, Nunes P, Wuthier RE (2007). Inhibitory effects of fusarochromanone on melanoma growth. Anticancer Drugs.

[13] Minervini F, Lucivero G, Visconti A, Bottalico C (1992). Immunomodulatory effects of fusarochromanones TDP-1 and TDP-2. Nat Toxins.

[14] Mahdavian E, Marshall M, Martin PM, Cagle P, Salvatore BA, Quick QA (2014). Caspase-dependent signaling underlies glioblastoma cell death in response to the fungal metabolite, fusarochromanone. Int J Mol Med.

[15] Mahdavian E, Palyok P, Adelmund S, Williams-Hart T, Furmanski BD, Kim YJ, Gu Y, Barzegar M, Wu Y, Bhinge KN, Kolluru GK, Quick Q, Liu YY, Kevil CG, Salvatore BA, Huang S, Clifford JL (2014). Biological activities of fusarochromanone: a potent anti-cancer agent. BMC Res Notes.

[16] Gu Y, Chen X, Shang C, Singh K, Barzegar M, Mahdavian E, Salvatore BA, Jiang S, Huang S (2014). Fusarochromanone induces G1 cell cycle arrest and apoptosis in COS7 and HEK293 cells. PLoS One.

[17] Shen HM, Shi CY, Shen Y, Ong CN (1996). Detection of elevated reactive oxygen species level in cultured rat hepatocytes treated with aflatoxin B1. Free Radic Biol Med.

[18] Stockmann-Juvala H, Mikkola J, Naarala J, Loikkanen J, Elovaara E, Savolainen K (2004). Oxidative stress induced by fumonisin B1 in continuous human and rodent neural cell cultures. Free Radic Res.

[19] Guerra MC, Galvano F, Bonsi L, Speroni E, Costa S, Renzulli C, Cervellati R (2005). Cyanidin-3-O-beta-glucopyranoside, a natural free-radical scavenger against aflatoxin B1- and ochratoxin A-induced cell damage in a human hepatoma cell line (Hep G2) and a human colonic adenocarcinoma cell line (CaCo-2). Br J Nutr.

[20] Bouaziz C, Sharaf El Dein O, El Golli E, Abid-Essefi S, Brenner C, Lemaire C, Bacha H (2008). Different apoptotic pathways induced by zearalenone, T-2 toxin and ochratoxin A in human hepatoma cells. Toxicology.

[21] Wu TS, Liao YC, Yu FY, Chang CH, Liu BH (2008). Mechanism of patulin-induced apoptosis in human leukemia cells (HL-60). Toxicol Lett.

[22] Krishnaswamy R, Devaraj SN, Padma VV (2010). Lutein protects HT-29 cells against Deoxynivalenol-induced oxidative stress and apoptosis: prevention of NF-κB nuclear localization and down regulation of NF-κB and Cyclo-Oxygenase-2 expression. Free Radic Biol Med.

[23] Fang H, Wu Y, Guo J, Rong J, Ma L, Zhao Z, Zuo D, Peng S (2012). T-2 toxin induces apoptosis in differentiated murine embryonic stem cells through reactive oxygen species-mediated mitochondrial pathway. Apoptosis.

[24] Cui J, Liu J, Wu S, Wang Y, Shen H, Xing L, Wang J, Yan X, Zhang X (2013). Oxidative DNA damage is involved in ochratoxin A-induced G2 arrest through ataxia telangiectasia-mutated (ATM) pathways in human gastric epithelium GES-1 cells *in vitro*. Arch Toxicol.

[25] Yang Q, He X, Li X, Xu W, Luo Y, Yang X, Wang Y, Li Y, Huang K (2014). DNA damage and S phase arrest induced by Ochratoxin A in human embryonic kidney cells (HEK 293). Mutat Res.

[26] Boussabbeh M, Ben Salem I, Prola A, Guilbert A, Bacha H, Abid-Essefi S, Lemaire C (2015). Patulin induces apoptosis through ROS-mediated endoplasmic reticulum stress pathway. Toxicol Sci.

[27] Ray PD, Huang BW, Tsuji Y (2012). Reactive oxygen species (ROS) homeostasis and redox regulation in cellular signaling. Cell Signal.

[28] Stadtman ER (1992). Protein oxidation and aging. Science.

[29] Chen L, Liu L, Yin J, Luo Y, Huang S (2009). Hydrogen peroxide-induced neuronal apoptosis is associated with inhibition of protein phosphatase 2A and 5, leading to activation of MAPK pathway. Int J Biochem Cell Biol.

[30] Chen L, Liu L, Huang S (2008). Cadmium activates the mitogen-activated protein kinase (MAPK) pathway via induction of reactive oxygen species and inhibition of protein phosphatases 2A and 5. Free Radic Biol Med.

[31] Kyriakis JM, Avruch J (2012). Mammalian MAPK signal transduction pathways activated by stress and inflammation: a 10-year update. Physiol Rev.

[32] Kim EK, Choi EJ (2010). Pathological roles of MAPK signaling pathways in human diseases. Biochim Biophys Acta.

[33] Wang X, Pesakhov S, Harrison JS, Kafka M, Danilenko M, Studzinski GP (2015). The MAPK ERK5, but not ERK1/2, inhibits the progression of monocytic phenotype to the functioning macrophage. Exp Cell Res.

[34] Huang S, Shu L, Easton J, Harwood FC, Germain GS, Ichijo H, Houghton PJ (2004). Inhibition of mammalian target of rapamycin activates apoptosis signal-regulating kinase 1 signaling by suppressing protein phosphatase 5 activity. J Biol Chem.

[35] Franklin CC, Kraft AS (1997). Conditional expression of the mitogen-activated protein kinase (MAPK) phosphatase MKP-1 preferentially inhibits p38 MAPK and stress-activated protein kinase in U937 cells. J Biol Chem.

[36] Morita K, Saitoh M, Tobiume K, Matsuura H, Enomoto S, Nishitoh H, Ichijo H (2001). Negative feedback regulation of ASK1 by protein phosphatase 5 (PP5) in response to oxidative stress. EMBO J.

[37] Zhao WQ, Feng C, Alkon DL (2003). Impairment of phosphatase 2A contributes to the prolonged MAP kinase phosphorylation in Alzheimer's disease fibroblasts. Neurobiol Dis.

[38] Aguilar JL, Kulkarni R, Randis TM, Soman S, Kikuchi A, Yin Y, Ratner AJ (2009). Phosphatase-dependent regulation of epithelial mitogen-activated protein kinase responses to toxin-induced membrane pores. PLoS One.

[39] Janssens V, Goris J (2001). Protein phosphatase 2A: a highly regulated family of serine/threonine phosphatases implicated in cell growth and signalling. Biochem J.

[40] Kowluru A, Seavey SE, Rabaglia ME, Nesher R, Metz SA (1996). Carboxylmethylation of the catalytic subunit of protein phosphatase 2A in insulin-secreting cells: evidence for functional consequences on enzyme activity and insulin secretion. Endocrinology.

[41] Chen J, Martin BL, Brautigan DL (1992). Regulation of protein serine-threonine phosphatase type-2A by tyrosine phosphorylation. Science.

[42] Thannickal VJ, Fanburg BL (2000). Reactive oxygen species in cell signaling. Am J Physiol Lung Cell Mol Physiol.

[43] Morrison E, Rundberget T, Kosiak B, Aastveit AH, Bernhoft A (2002). Cytotoxicity of trichothecenes and fusarochromanone produced by Fusarium equiseti strains isolated from Norwegian cereals. Mycopathologia.

[44] Nusuetrong P, Yoshida M, Tanitsu MA, Kikuchi H, Mizugaki M, Shimazu K, Pengsuparp T, Meksuriyen D, Oshima Y, Nakahata N (2005). Involvement of reactive oxygen species and stress-activated MAPKs in satratoxin H-induced apoptosis. Eur J Pharmacol.

[45] Shen H, Liu J, Wang Y, Lian H, Wang J, Xing L, Yan X, Wang J, Zhang X (2013). Aflatoxin G1-induced oxidative stress causes DNA damage and triggers apoptosis through MAPK signaling pathway in A549 cells. Food Chem Toxicol.

[46] Agrawal M, Bhaskar AS, Lakshmana Rao PV (2015). Involvement of mitogen-activated protein kinase pathway in T-2 toxin-induced cell cycle alteration and apoptosis in human neuroblastoma cells. Mol Neurobiol.

[47] Lee J, Chen Y, Tolstykh T, Stock J (1996). A specific protein carboxyl methylesterase that demethylates phosphoprotein phosphatase 2A in bovine brain. Proc Natl Acad Sci USA.

[48] De Baere I, Derua R, Janssens V, Van Hoof C, Waelkens E, Merlevede W, Goris J (1999). Purification of porcine brain protein phosphatase 2A leucine carboxyl methyltransferase and cloning of the human homologue. Biochemistry.

[49] Xing Y, Li Z, Chen Y, Stock JB, Jeffrey PD, Shi Y (2008). Structural mechanism of demethylation and inactivation of protein phosphatase 2A. Cell.

[50] Chen J, Parsons S, Brautigan DL (1994). Tyrosine phosphorylation of protein phosphatase 2A in response to growth stimulation and v-src transformation of fibroblasts. J Biol Chem.

[51] Trachootham D, Alexandre J, Huang P (2009). Targeting cancer cells by ROS-mediated mechanisms: a radical therapeutic approach?. Nat Rev Drug Discov.

[52] Han X, Xu B, Beevers CS, Odaka Y, Chen L, Liu L, Luo Y, Zhou H, Chen W, Shen T, Huang S (2012). Curcumin inhibits protein phosphatases 2A and 5, leading to activation of mitogen-activated protein kinases and death in tumor cells. Carcinogenesis.

[53] Xu C, Zhang H, Liu C, Zhu Y, Wang X, Gao W, Huang S, Chen L (2015). Rapamycin inhibits Erk1/2-mediated neuronal apoptosis caused by cadmium. Oncotarget.

[54] Zhou H, Shen T, Shang C, Luo Y, Liu L, Yan J, Li Y, Huang S (2014). Ciclopirox induces autophagy through reactive oxygen species-mediated activation of JNK signaling pathway. Oncotarget.

